# Evidence for antibody as a protective correlate for COVID-19 vaccines

**DOI:** 10.1016/j.vaccine.2021.05.063

**Published:** 2021-07-22

**Authors:** Kristen A. Earle, Donna M. Ambrosino, Andrew Fiore-Gartland, David Goldblatt, Peter B. Gilbert, George R. Siber, Peter Dull, Stanley A. Plotkin

**Affiliations:** a Vaccine Development & Surveillance, Bill & Melinda Gates Foundation, 500 5th Ave N, Seattle, WA 98109, USA; b Independent Advisor, Stuart, FL, USA; c Vaccine and Infectious Disease Division, Fred Hutchinson Cancer Research Center, 1100 Fairview Ave N, Seattle, WA 98109, USA; d Great Ormond Street Institute of Child Health, University College London, 30 Guilford Street, London WC1N 1EH, UK; e Independent Advisor, New York, NY, USA; f Department of Pediatrics, Perelman School of Medicine, University of Pennsylvania, 3401 Civic Center Blvd., Philadelphia, PA 19104, USA

**Keywords:** COVID-19, SARS-CoV-2, Vaccine, Correlate of protection

## Abstract

A correlate of protection (CoP) is urgently needed to expedite development of additional COVID-19 vaccines to meet unprecedented global demand. To assess whether antibody titers may reasonably predict efficacy and serve as the basis of a CoP, we evaluated the relationship between efficacy and in vitro neutralizing and binding antibodies of 7 vaccines for which sufficient data have been generated. Once calibrated to titers of human convalescent sera reported in each study, a robust correlation was seen between neutralizing titer and efficacy (ρ = 0.79) and binding antibody titer and efficacy (ρ = 0.93), despite geographically diverse study populations subject to different forces of infection and circulating variants, and use of different endpoints, assays, convalescent sera panels and manufacturing platforms. Together with evidence from natural history studies and animal models, these results support the use of post-immunization antibody titers as the basis for establishing a correlate of protection for COVID-19 vaccines.

## 1 Introduction

Eleven novel COVID-19 vaccines have demonstrated efficacy with several more undergoing Phase III clinical trials. Despite this, to meet unprecedented global demand, additional vaccines are needed even as placebo-controlled efficacy trials are becoming infeasible [1]. An immunological correlate of protection (CoP) is urgently needed not only to provide a path for regulatory approval of new scalable, deliverable, and affordable vaccines, but for a number of other applications, including: Phase IV studies that enable the most efficient use of approved vaccines (i.e., heterologous priming and prime-boost regimens); serosurveys to evaluate protection levels of populations; and to assist in predicting the durability of protection. Many vaccines have been licensed or had expanded indications based on a binding or functional antibody CoP established in multiple efficacy trials [2], but for COVID-19 these subject level data analyses and a consensus around the threshold of protection across multiple studies and populations are unavailable. Meanwhile, a mounting body of evidence from non-human primate [3] and natural history [4] studies suggests that an antibody-based correlate of protection can be estimated for COVID-19 vaccines. We therefore assessed the relationship between the efficacy of seven COVID-19 vaccines in Phase III trials and the levels of both virus neutralizing antibody (VNA) and Spike protein-binding IgG antibody to determine whether either assay may serve as a predictor of vaccine efficacy against COVID-19.

## 2 Methods

### 2.1 Data selection

Inclusion criteria for immunogenicity and vaccine efficacy data are described in the Supplemental Appendix. At the time of analysis, seven vaccines met these criteria: Pfizer, Moderna, Gamaleya, AstraZeneca, Sinovac, Novavax, and Johnson & Johnson.

### 2.2 Statistical analysis

Vaccine efficacy (VE) was computed as one minus risk-ratio times 100, and the risk-ratio for each study was calculated as specified in the study protocol/primary publication. Correlation was the Spearman's rank correlation coefficient (ρ) between the readouts on the x- and y-axes; both x and y data were fit using a natural log transform. The dashed fit line was computed using locally estimated scatterplot smoothing (LOESS) regression (all points fit, with tricube weight function). We applied a non-parametric Bayesian approach to evaluate the quality of VNA and binding antibodies as trial-level surrogate endpoints [5]. Leave-one-out cross-validation was applied to evaluate how well VE in each held-out trial could be predicted from the observed biomarker distribution and the model from the six other trials linking geometric mean biomarker level to VE.

## 3 Results

**Fig. 1:**
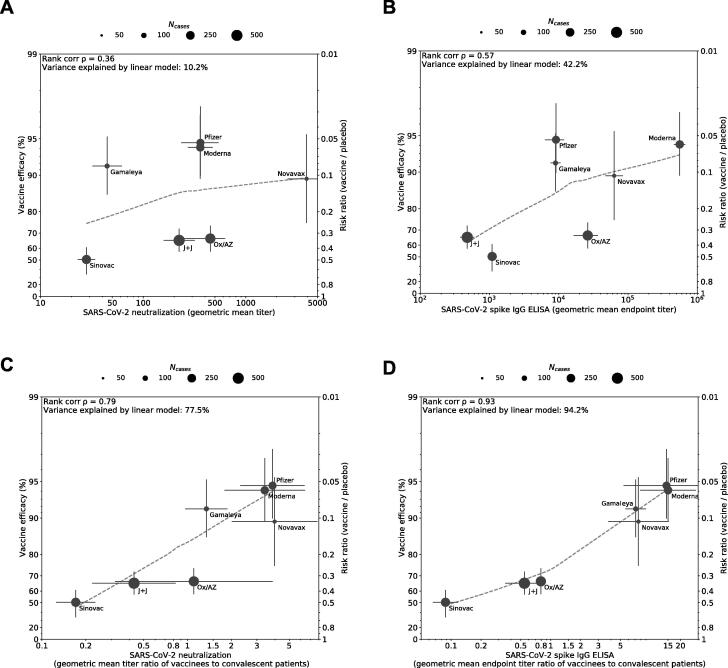
**Correlation between antibody responses and efficacy rate for 7 COVID-19 vaccines.** Panels A and B display correlations of antibody responses for neutralization and ELISA assay ratios, respectively, without HCS calibration. Panels C and D display the same vaccine-induced responses, but with HCS calibration. Data included in correlation analyses are described in Tables S1 and S2. Dot size corresponds to the number of cases reported for Phase III efficacy analyses. The y-axis is estimated log risk ratio reported on the vaccine efficacy scale. The x-axis is ratio of the peak geometric mean neutralization titer or ELISA titer at 7–28 days post vaccination, relative to HCS. Error bars indicate 95% confidence Intervals (except for Oxford/AZ antibody responses, which represent ratios of median titers with interquartile ranges) with dashed line showing non-parametric LOESS fit. A rank correlation value was calculated with R^2^ in a linear model utilized for variance explanation.

**Fig. 2:**
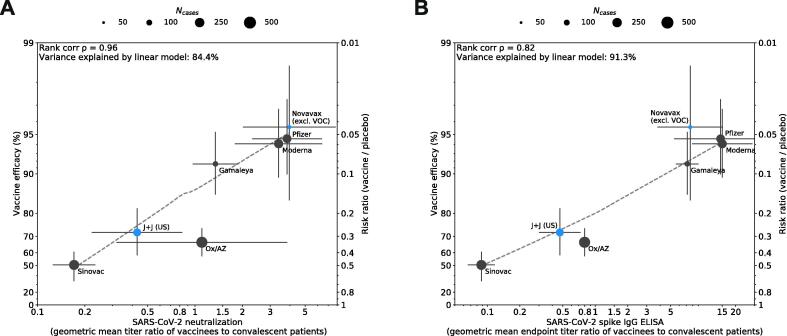
**Impact of post-hoc analyses to assess efficacy against ancestral strain on correlation.** To assess the impact that variation in circulating strains may have on correlation between neutralizing antibody titer (Panel A) or binding antibody titer (Panel B) and efficacy, post-hoc analyses that calculate efficacy against the dominant ancestral strain D614G (Novavax) or calculate efficacy at sites without circulating VOCs (Janssen/J&J) were substituted for primary endpoint efficacy estimates (blue dots). Binding antibody ratio for J&J (Panel B) was calculated from US-specific Phase III data, calibrated to HCS titers published with Phase I/II immunogenicity data, as noted in Table S2.

**Fig. 3:**
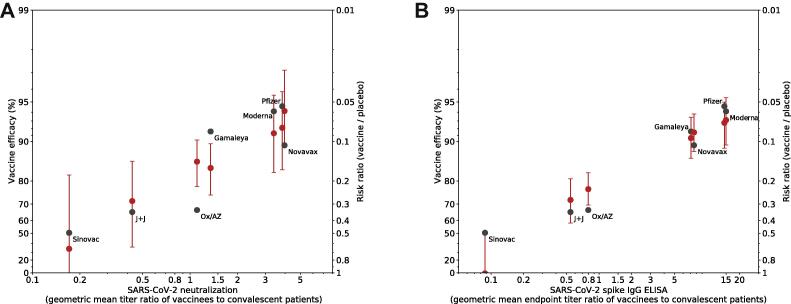
**Impact of exploratory analysis of Oxford/AstraZeneca immunogenicity and efficacy by interval between doses on correlation.** Publication of a pooled analysis of Phase 3 trial data for Oxford/AstraZeneca suggests that interval between doses in Phase 3 studies varied from 4 to 12+ weeks, and that both vaccine efficacy and immunogenicity data varied by dose interval. The immunogenicity data from Phase 1/2, which corresponded to a 4-week dose interval, may therefore not be representative of immunogenicity generated in the Phase 3 study, or correspond to pooled vaccine efficacy estimates. To assess the impact on correlation, exploratory analyses of dose intervals < 6 week and ≥ 12 weeks, and corresponding immunogenicity and efficacy, were substituted for the pooled vaccine efficacy estimate used in Fig. 1. Exploratory analyses are denoted by blue dots. Ratios for neutralizing antibody titer (Panel A) and binding antibody titer (Panel B) were generated for Oxford/AstraZeneca using the HCS median titer from Phase 1/2 publication, as noted in Tables S1 and S2.

We first evaluated peak geometric mean titers (GMT) of VNA and binding antibodies 1–4 weeks following the recommended vaccination regimen as reported by each manufacturer but found low correlations with efficacy (Fig. 1A, 1B), most likely because assays were not calibrated to a common standard. We then calibrated assays against an imperfect but “best available” standard, titers of human convalescent serum (HCS) reported in each study, to generate a vaccinated:convalescent sera ratio; this revealed high correlation between the VNA ratio and efficacy (ρ = 0.79) and binding antibody titer ratio and efficacy (ρ = 0.93) (Fig. 1C, 1D). Neutralizing or IgG binding antibody accounted for 77.5% and 94.2%, respectively, of the variation in efficacy observed among the seven vaccines. To assess the impact that circulating variants may have on this relationship, we substituted primary endpoint efficacy estimates with post-hoc analyses that either calculate efficacy against the wildtype, D614G strain of SARS-CoV-2 (Novavax) or calculate efficacy at sites without significant representation of circulating variants (Janssen/J&J’s U.S. sites) where available. Post-hoc analysis of Novavax vaccine efficacy against the ancestral strain (95.6%) was determined by sequencing 56 of the 62 cases accumulated in the UK Phase III study [6]. Vaccine efficacy for the U.S. sites of Janssen/J&J’s Phase III study (72%) is included based on sequencing of 197 of the 268 cases, suggesting that strain D614G accounted for the vast majority (96.4%) of cases [7]. Controlling for efficacy against the ancestral strain strengthened the correlation between VE and the VNA ratio (ρ = 0.96, Fig. 2A), but weakened the correlation between VE and binding titer ratios (ρ = 0.82, Fig. 2B). Furthermore, accounting for increased antibody responses associated with an extended interval schedule for the Oxford/AstraZeneca vaccine improved the correlation between VE and both ratios (ρ = 0.86, ρ = 0.93, Fig. 3).

**Fig. 4:**
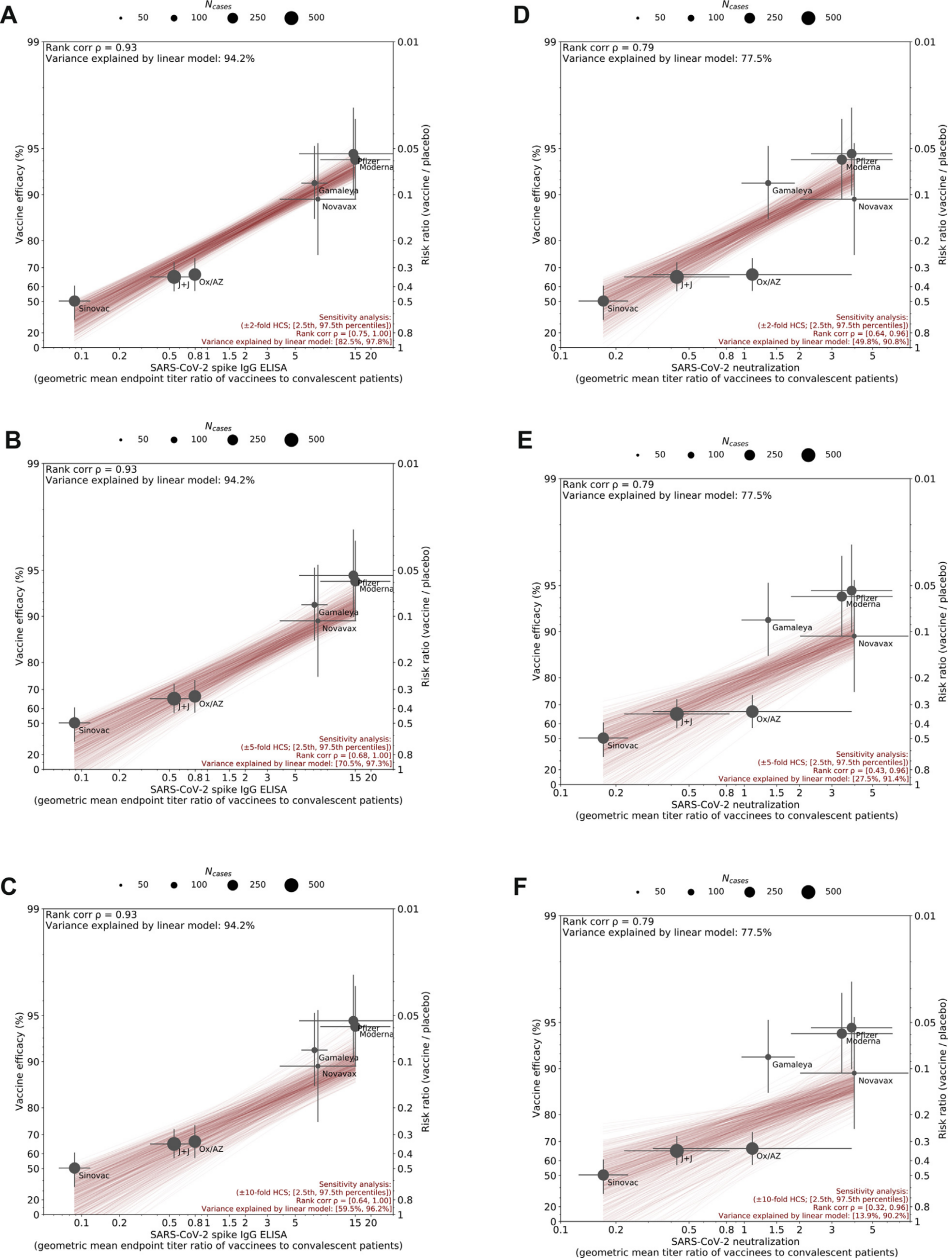
**Prediction of vaccine efficacy using a cross-validation leave-one-out analysis.** A non-parametric Bayesian approach was used to evaluate antibody titers as a potential correlate. Neutralizing antibody titer (A) and binding antibody titer (B) were each evaluated using leave-one-out cross-validation: the immune response and VE from six of the trials was used to build a model to predict the VE in the seventh trial based on the observed immune response distribution. Predicted VE is shown for each trial (red dots and 95% confidence interval). Observed VE and immune response data are also plotted for reference (black dots).

Using a non-parametric approach, we modeled the relationship of each of the candidate biomarkers with VE across the seven vaccines and attempted to predict the VE of each vaccine based on its biomarker response (Methods). For both the VNA ratios (Fig. 4A) and binding antibody ratios (Fig. 4B) the observed VE fell within the 95% credible interval of the model’s prediction for all vaccines, with the exception of Oxford/AstraZeneca and Gamaleya for VNA and Sinovac for binding antibodies.

## 4 Discussion

In this study, a robust correlation was seen between antibody titers and efficacy across seven different vaccines, with higher titers correlating with higher vaccine efficacy, despite uncontrolled variables across the studies. First, these vaccines represent four vaccine manufacturing platforms with varying levels of engagement of humoral and cell-mediated immunity: mRNA, adenoviral vector, protein subunit, and inactivated virus vaccines. Second, our analysis relies upon calibration to HCS panels selected by each developer, which vary widely in sample size and representation of the range of disease severities. As both neutralizing and binding antibody responses to natural infection correlate significantly with severity of disease, the specific HCS panel selected may significantly impact ratios generated in our analysis [8], [9]; however, sensitivity analysis suggested that the correlations were robust even to randomly generated 2–10 fold shifts in the HCS geometric mean values (Fig. S1). As expected, when the shifts are increased the range of correlations becomes broader and approaches the uncalibrated correlations.

Third, these clinical studies were conducted on geographically diverse study populations, which were subject to different forces of infection and circulating variants. Variability in the timing and location of the Phase III efficacy studies included in our analysis suggests that the efficacy point estimates will be differentially impacted by circulating variants of concern (VOCs) which are more likely to escape protection afforded by vaccination [10], which is supported by the increased correlation when isolating VE against the wildtype strain (Fig. 2A). Finally, this analysis is subject to variability in clinical protocols, including endpoints, case definitions, and assays, and in execution of these protocols. Our analyses suggest that the correlation between efficacy and antibody biomarkers is improved by including efficacy point estimates that most closely correspond to the dose and schedule included in the Phase I/II immunogenicity study (Fig. 3), and we anticipate that this relationship will be further elucidated once immunogenicity data are generated using a common assay and calibrated to the International Standard. Finally, the observation that most of the predicted VE 95% Cis include the observed VE (Fig. 4) supports the hypothesis that VNA and/or ELISA may be good correlates of protection. Despite these many caveats, a robust correlation between antibody titers and efficacy is demonstrated in these results, and these results support the utility of the antibody biomarkers for predicting VE in new settings and for novel COVID-19 vaccines currently in development.

As additional data have become available, three issues challenge the interpretation and application of this model to further vaccine development. First, trials of several vaccines (AstraZeneca, Moderna, Pfizer) have shown substantial efficacy following the first dose despite low neutralization titers, with many subjects with titers from the pre-dose two sample below the lower limit of quantification. This suggests either relatively low neutralization assay sensitivity or non-neutralizing antibodies and T cell responses may be functionally important, which is supported by the evidence that binding antibody titers appear robust at the same post-Dose 1 time points [11], [12] and that Fc functional antibody responses play a role in recovery from natural infection [13] and correlate with protective efficacy [3]. Nonetheless, virus neutralization assays remain an attractive candidate for the basis of a correlate, as they represent a functional assay with high likelihood of predicting efficacy following the full vaccination regimen. Second, the rise in prominence of VOCs raises questions about the projected efficacy of many candidates currently in development. Predicting efficacy against VOCs will require measuring neutralization titers to VOCs. While we do not yet have the data needed to validate whether this model may be predictive for efficacy against specific variants, it is encouraging that data available to date suggest that consistent with this paradigm, reduction in neutralization of B.1.351 by vaccinated sera corresponds to a reduction in efficacy [10], [14]. Third, our meta-analysis evaluated prediction of vaccine efficacy over 3–5-month follow-up periods, with data for predicting efficacy durability not yet available. Declining neutralization titers with time may correlate with or cause falling efficacy, perhaps requiring boosters.

The results reported here support the use of post-immunization antibody levels as the basis for a CoP. We propose that the next steps toward achieving consensus on a CoP include the following: First, to establish comparability of antibody measurements among laboratories by (i) using the WHO International Standard (NIBSC 20/136) to express VNA titers in IU/ml and binding antibody titers in BAU/ml and (ii) establishing a relevant proficiency panel. Second, to agree on an assay, most likely a neutralization assay meeting performance-based criteria, to serve as the gold standard assay for CoP and perhaps to allow validation of secondary assays by strong correlations with the gold standard. Third, where possible, to calculate the protective threshold in each Phase III study based either on the distribution of antibody titers in random samples of vaccinees and controls [15] or on case-cohort evaluations [16]. Fourth, since the CoP calculated from different studies may differ, stakeholders should be convened to arrive at a consensus on a minimum protective antibody level for the primary outcome of symptomatic COVID. If possible, it would also be useful to estimate thresholds for preventing severe disease or asymptomatic infection. Fifth, to verify that the CoP will apply to new variants using appropriate adapted assays as information accrues on the immune response and efficacy of vaccines against them.

A CoP agreed to by regulators will support the demonstration of efficacy of new vaccines using traditional criteria for establishing non-inferiority based on the proportion of vaccinees achieving the protective threshold and comparable GMTs, relative to an approved comparator vaccine. Since the relationship between antibody responses and efficacy was consistent across all manufacturing platforms evaluated to date, even though they may differ in their induction of other antibody functions or T cell responses, an argument can be made that any approved vaccine can serve as a comparator for a future candidate vaccine. Even if authorized for use based on an immunologic readout, full licensure should require confirmatory effectiveness evaluations to closely follow any conditional or accelerated approval process.

## Funding

This research did not receive any specific grant from funding agencies in the public, commercial, or not-for-profit sectors.

## Declaration of Competing Interest

The authors declare the following financial interests/personal relationships which may be considered as potential competing interests: Dr. Plotkin consults for Janssen and Moderna; Dr. Siber reports personal fees from Clover Biopharmaceuticals, other from COVAXX, personal fees from CanSino, personal fees from CureVac, personal fees from Valneva, personal fees and other from Affinivax, outside the submitted work; Dr. Gilbert reports grants and non-financial support from SanofiPasteur, outside the submitted work; Dr. Ambrosino reports personal fees from COVAXX, personal fees from Clover Biopharmaceuticals, outside the submitted work.
