# Supplementary material for: Evidence for antibody as a protective correlate for COVID-19 vaccines
Source: Vaccine. 2021 Jul 22;39(32):4423–8. doi: 10.1016/j.vaccine.2021.05.063 (PMC8142841; doi:10.1016/j.vaccine.2021.05.063)
Supplement: Supplementary data 1 [file mmc1.docx]

Supplementary Appendix

**Table of Contents**

Methods

Immunogenicity data………………………………………………………………………………………………………………………..3

Vaccine efficacy……………………………..……………………………………………..….………………………………………………3

Statistical analysis……………………………………………………………………………………………………………………………..3

Supplementary figures

Figure S1: Sensitivity analyses modeling impact of 2-10 fold changes in HCS values………………………….4

Tables

Table S1: Data summary and sources for vaccine-induced and HCS neutralizing antibody titers……....5

Table S2: Data summary and sources for vaccine-induced and HCS binding antibody titers………………7

References…………………………………………………………………………………………………….…………………………………………9

Methods

Immunogenicity data

Neutralizing and binding antibody titers were identified from peer-reviewed publications of Phase 1 or Phase 2 clinical studies for inclusion in the primary analysis. Publication of a human convalescent sera (HCS) panel with Phase 1/2 immunogenicity data was required for inclusion in the analysis. When data was not presented in a table it was obtained from a figure using WebPlotDigitizer (18). Where multiple regimens or post-vaccination timepoints were published, the data points that correspond to peak response in the 1-4 weeks following vaccination according to the dose and schedule studied in Phase 3 were chosen. In cases where multiple assays were employed to describe immunogenicity, the assay with the most comprehensive HCS panel run alongside subject data was selected.

Additional publicly available regulatory documents and manuscript pre-prints were considered for evaluation in exploratory analyses included in the supplement. Data summary and sources are provided in Tables S1 and S2.

Neutralization antibody geometric mean titers were generated from wild-type virus neutralization assays for all vaccines except Moderna and AstraZeneca / Oxford, where pseudoviral-based assays were utilized. Anti-SARS-CoV-2 binding titers were generated by anti-Spike protein ELISAs, except for Moderna, Gamaleya, and Sinovac, which utilized an anti-Receptor Binding Domain ELISA, and Pfizer/BioNTech which utilized an anti-S1 ELISA.

Vaccine efficacy

COVID-19 vaccines were considered for inclusion in the correlation analysis if interim or primary analysis efficacy point estimates were publicly available at the time of submission from at least one comprehensive source (i.e., peer-reviewed publication, regulatory submission, or release of detailed site- or strain-level data within a press release or briefing). Primary endpoint efficacy analyses were employed to conduct the primary correlation analysis for this study. Post hoc efficacy estimates were considered for evaluation in exploratory analyses included in the supplement.

Statistical analyses

As a sensitivity analysis we estimated rank correlation coefficients, percent variance explained and linear fit lines from VNA (ELISA) ratios that were simulated using HCS VNA (ELISA) geometric means that were randomly shifted from the observed geometric means. The random shifts were simulated using uniform sampling over +/- 2-fold 5-fold or 10-fold range. Datasets were simulated 10K times to estimate the 2.5th and 97.5th percentiles of the rank correlation and percent variance explained; fit lines from 500 simulations were plotted in the figure.

Supplementary correlates analyses were computed using identical methods, but with alternative data points (blue) indicated in the figure legend.

**Figure S1. Sensitivity analyses simulating random variation in HCS values.** To assess the sensitivity of this analysis to the HCS panel selected by each developer, rank correlation coefficients, percent variance explained and linear fit lines were estimated from VNA (A-C) and ELISA (D-F) ratios that were simulated using HCS geometric means that were randomly shifted the observed geometric means. The random shifts were simulated using uniform sampling over +/- 2-fold range (A, D), a +/- 5-fold range (B, E) or +/ 10-fold range (C, F). Datasets were simulated 10K times to estimate the 2.5th and 97.5th percentiles of the rank correlation and percent variance explained; fit lines from 500 simulations are plotted.


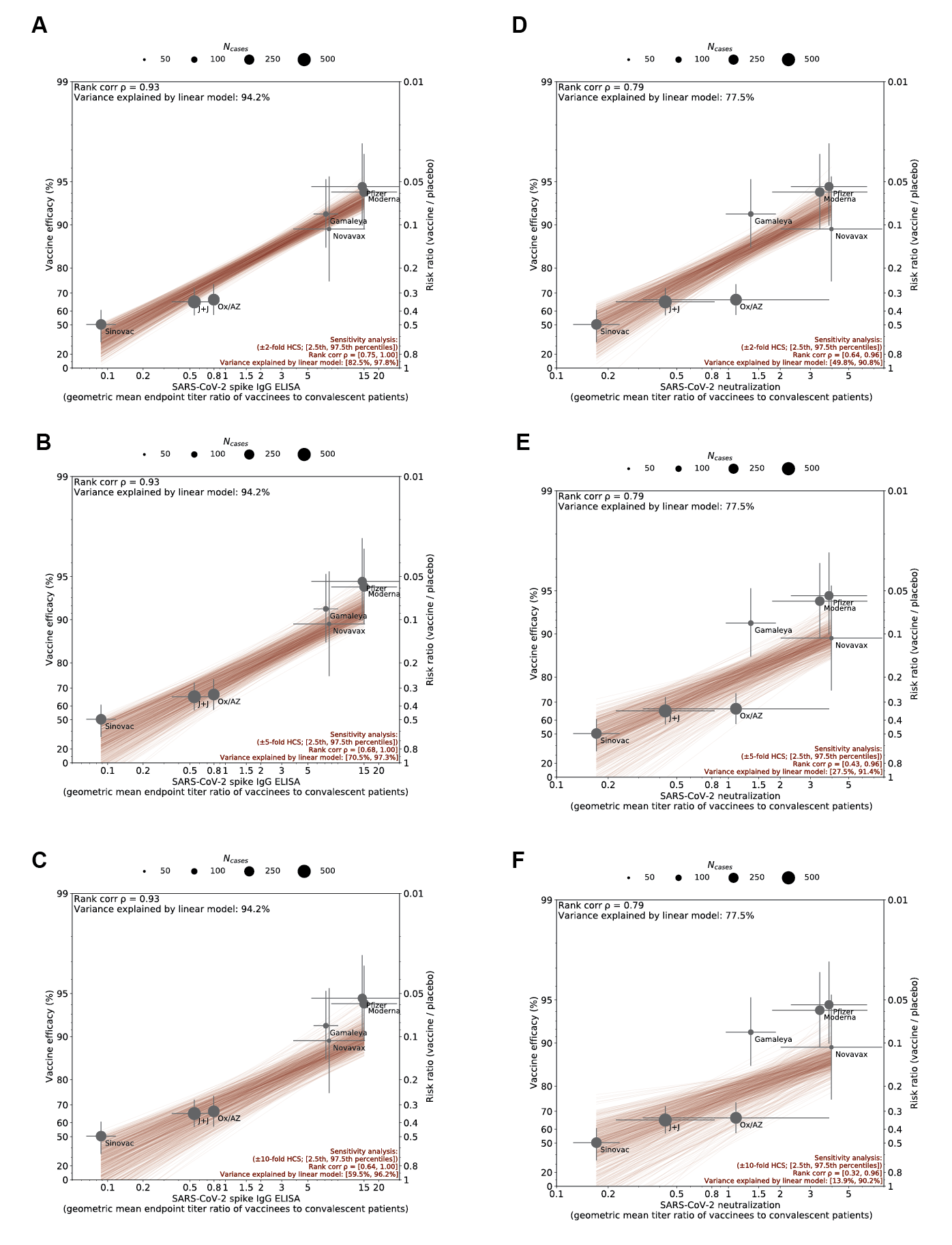


**Table S1. Data summary and sources for vaccine-induced and HCS neutralizing antibody titers.**

| **Developer** | **% Efficacy (CI)** | **Cases (N)** | **Vaccine-induced** | |  | **HCS** | |  |  |  |
| --- | --- | --- | --- | --- | --- | --- | --- | --- | --- | --- |
|  |  |  | **GMT (CI)** | **N** |  | **GMT (CI)** | **N** | **Vaccine/**  **HCS ratio** | **Assay** | **HCS description** |
| **Pfizer/BioNTech** |  |  |  |  |  |  |  |  |  |  |
| Polack et al.^3^ | 94.6 (89.9, 97.3) | 178 |  |  |  |  |  |  |  |  |
| Walsh et al.^4^ |  |  | 361 (235, 541†) | 11 |  | 94 (61, 110†) | 38 | 3.8 | WT VNA | Asymptomatic + symptomatic (mild-severe) |
| **Moderna** |  |  |  |  |  |  |  |  |  |  |
| Baden et al.^5^ | 94.1 (89.3, 96.8) | 196 |  |  |  |  |  |  |  |  |
| Anderson et al.^6^ |  |  | 360 (273, 476) | 14 |  | 106 (60, 189) | 41 | 3.4 | Ps VNA | Symptomatic (mild-severe) |
| **Gamaleya** |  |  |  |  |  |  |  |  |  |  |
| Logunov et al. (2021)^7^ | 91.6 (85.6, 95.2) | 78 | 44.5 (32, 62) | 100 |  |  |  | 1.4 | WT VNA |  |
| Logunov et al. (2020)^8^ |  |  |  |  |  | 33 (31.5, 34.5) | 4817 |  | WT VNA | Symptomatic (mild-moderate) |
| **Oxford/AstraZeneca (Primary correlation analysis)** | | | | | | | | |  |  |
| Voysey et al.^2^ | 66.7 (57.4, 74.0) | 332 |  |  |  |  |  |  |  |  |
| Folegatti et al.^9^ |  |  | 451 (212, 628)* | 9 |  | 408 (212, 2000) *† | 170 | 1.1 | Ps VNA | Asymptomatic + symptomatic (mild-severe) |
| **Sinovac** |  |  |  |  |  |  |  |  |  |  |
| ANVISA press briefing^10^ | 50.4 (34.1, 60.7) | 252 |  |  |  |  |  |  |  |  |
| Zhang et al.^11^ |  |  | 28 (23, 34) | 118 |  | 164 (129, 209) | 117 | 0.17 | WT VNA | Symptomatic (mild-severe) |
| **Novavax (Primary correlation analysis)** | | | |  |  |  |  |  |  |  |
| Novavax press briefing^12^ | 89.3 (75.2, 95.4) | 62 |  |  |  |  |  |  |  |  |
| Keech et al.^13^ |  |  | 3906 (2556, 5970) | 29 |  | 984 (579, 1671) | 32 | 4.0 | WT VNA | Asymptomatic + symptomatic (majority mild) |
| **Janssen/J&J (Primary correlation analysis)** | | | | |  |  |  |  |  |  |
| FDA briefing document^14^ | 65.5 (57.2, 72.4) | 437 |  |  |  |  |  |  |  |  |
| Sadoff et al.^15^ |  |  | 224 (158, 318) | 24 |  | 522 (277, 850)† | 32 | 0.43 | WT VNA | Symptomatic (majority severe) |
| **Novavax (Excl. VOC, Fig. 2)** | | | | | | |  |  |  |  |
| Novavax press briefing^12^ | 95.6 (84.1, 98.5) | 56 |  |  |  |  |  |  |  |  |
| Keech et al.^13^ |  |  | 3906 (2556, 5970) | 29 |  | 984 (579, 1671) | 32 | 4.0 | WT VNA | Asymptomatic + symptomatic (majority mild) |
| **Janssen/J&J (US, Fig. 2)** | | | | | |  |  |  |  |  |
| FDA briefing document^16^ | 72.0 (58.2, 81.7) | 144 |  |  |  |  |  |  |  |  |
| Sadoff et al.^15^ |  |  | 224 (158, 318) † | 24 |  | 522 (277, 850) † | 32 | 0.43 | WT VNA | Symptomatic (majority severe) |
| **Oxford/AstraZeneca (< 6wks, Fig. 3)** | | | | | | | | | | |
| Voysey et al.^2^ | 54.9 (32.7, 69.7) | 111 | 125 (110, 141) | 272 |  |  |  | 0.31 | Ps VNA |  |
| Folegatti et al.^9^ |  |  |  |  |  | 408 (212, 2000) *† | 170 |  | Ps VNA | Asymptomatic + symptomatic (mild-severe) |
| **Oxford/AstraZeneca (≥ 12 wks, Fig. 3)** | | | | | | | | | | |
| Voysey et al.^2^ | 80.7 (66.5, 88.9) | 332 | 240 (210, 276) | 217 |  |  |  | 0.59 | Ps VNA |  |
| Folegatti et al.^9^ |  |  |  |  |  | 408 (212, 2000) *† | 170 |  | Ps VNA | Asymptomatic + symptomatic (mild-severe) |

WT VNA = Wild-type virus neutralization assay

Ps VNA = Pseudovirus neutralization assay

* median and inter-quartile range as published, instead of geometric mean titer and 95% CI

† Estimates were obtained from published figure

**Table S2. Data summary and sources for vaccine-induced and HCS binding antibody titers.**

| **Developer** | **% Efficacy (CI)** | **Cases (N)** | **Vaccine-induced** | |  | **HCS** | |  |  |  |
| --- | --- | --- | --- | --- | --- | --- | --- | --- | --- | --- |
|  |  |  | **GMT (CI)** | **N** |  | **GMT (CI)** | **N** | **Vaccine/**  **HCS ratio** | **ELISA antigen** | **HCS description** |
| **Pfizer/BioNTech** |  |  |  |  |  |  |  |  |  |  |
| Polack et al.^3^ | 94.6 (89.9, 97.3) | 178 |  |  |  |  |  |  |  |  |
| Walsh et al.^4^ |  |  | 9136 (6330, 11968†) | 11 |  | 631 (171, 1121†) | 38 | 15 | S1 | Asymptomatic + symptomatic (mild-severe) |
| **Moderna** |  |  |  |  |  |  |  |  |  |  |
| Baden et al.^5^ | 94.1 (89.3, 96.8) | 196 |  |  |  |  |  |  |  |  |
| Anderson et al.^6^ |  |  | 558,905 (462,907; 674,810) | 14 |  | 37,244 (20,170; 68,771) | 41 | 15 | RBD | Symptomatic (mild-severe) |
| **Gamaleya** |  |  |  |  |  |  |  |  |  |  |
| Logunov et al. (2021)^7^ | 91.6 (85.6, 95.2) | 78 | 8996 (7610; 10,635) | 100 |  |  |  | 7.1 | RBD |  |
| Logunov et al. (2020)^8^ |  |  |  |  |  | 1266 (1066, 1504) | 4817 |  | RBD | Symptomatic (mild-moderate) |
| **Oxford/AstraZeneca (Primary correlation analysis)** | | | | | | | | |  |  |
| Voysey et al.^2^ | 66.7 (57.4, 74.0) | 332 |  |  |  |  |  |  |  |  |
| Folegatti et al.^9^ |  |  |  |  |  | 33,000* (NA) | 180 | 0.80 | S | Asymptomatic + symptomatic (mild-severe) |
| Ramasamy et al.^17^ |  |  | 26,251* (16,453; 36,643) | 45 |  |  |  |  |  |  |
| **Sinovac** |  |  |  |  |  |  |  |  |  |  |
| ANVISA press briefing^10^ | 50.4 (34.1, 60.7) | 252 |  |  |  |  |  |  |  |  |
| Zhang et al.^11^ |  |  | 1094 (937, 1278) | 117 |  |  |  | 0.09 | RBD | Symptomatic (mild-severe) |
| Wang et al.^1^ |  |  |  |  |  | 12,442 (9755; 15,869) | 117 |  | RBD |  |
| **Novavax (Primary correlation analysis)** | | | |  |  |  |  |  |  |  |
| Novavax press briefing^12^ | 89.3 (75.2, 95.4) | 62 |  |  |  |  |  |  |  |  |
| Keech et al.^13^ |  |  | 63,160 (47,117; 84,666) | 29 |  | 8344 (4421; 15,748) | 32 | 7.6 | S | Asymptomatic + symptomatic (majority mild) |
| **Janssen/J&J (Primary correlation analysis)** | | | | |  |  |  |  |  |  |
| FDA briefing document^14^ | 65.5 (57.2, 72.4) | 437 |  |  |  |  |  |  |  |  |
| Sadoff et al.^15^ |  |  | 478 (379, 603) | 69 |  | 879 (628, 1332) | 32 | 0.54 | S | Symptomatic (majority severe) |
| **Novavax (Excl. VOC, Fig. 2)** | | | | | | |  |  |  |  |
| Novavax press briefing^12^ | 95.6 (84.1, 98.5) | 56 |  |  |  |  |  |  |  |  |
| Keech et al.^13^ |  |  | 63,160 (47,117; 84,666) † | 29 |  | 8344 (4421; 15,748) † | 32 | 7.6 | S | Asymptomatic + symptomatic (majority mild) |
| **Janssen/J&J (US, Fig. 2)** | | | | | |  |  |  |  |  |
| FDA briefing document^16^ | 72.0 (58.2, 81.7) | 144 | 412 (379, 603) | 48 |  |  |  | 0.47 | S |  |
| Sadoff et al.^15^ |  |  |  |  |  | 879 (628, 1332) † | 32 |  | S | Symptomatic (majority severe) |
| **Oxford/AstraZeneca (< 6wks, Fig. 3)** | | | | | | | | | | |
| Voysey et al.^2^ | 54.9 (32.7, 69.7) | 111 | 23,453 (21,040; 26,142) | 300 |  |  |  | 0.71 | S |  |
| Folegatti et al.^9^ |  |  |  |  |  | 33,000 (NA) † | 180 |  | S | Asymptomatic + symptomatic (mild-severe) |
| **Oxford/AstraZeneca (≥ 12 wks, Fig. 3)** | | | | | | | | | | |
| Voysey et al.^2^ | 80.7 (66.5, 88.9) | 332 | 47,942 (43,638; 52,670) | 397 |  |  |  | 1.5 | S |  |
| Folegatti et al.^9^ |  |  |  |  |  | 33,000 (NA) † | 180 |  | S | Asymptomatic + symptomatic (mild-severe) |

WT VNA = Wild-type virus neutralization assay

Ps VNA = Pseudovirus neutralization assay

NA = data not available

* median and inter-quartile range as published, instead of geometric mean titer and 95% CI

† Estimates obtained from published figure

**Supplemental References**

1 Wang X, Guo X, Xin Q, *et al.* Neutralizing Antibodies Responses to SARS-CoV-2 in COVID-19 Inpatients and Convalescent Patients. *Clin Infect Dis* 2020; **71**: 2688–94.

2 Voysey M, Clemens SAC, Madhi SA, *et al.* Single-dose administration and the influence of the timing of the booster dose on immunogenicity and efficacy of ChAdOx1 nCoV-19 (AZD1222) vaccine: a pooled analysis of four randomised trials. *The Lancet* 2021; **397**: 881–91.

3 Polack FP, Thomas SJ, Kitchin N, *et al.* Safety and Efficacy of the BNT162b2 mRNA Covid-19 Vaccine. *N Engl J Med* 2020; **383**: 2603–15.

4 Walsh EE, Frenck RW, Falsey AR, *et al.* Safety and Immunogenicity of Two RNA-Based Covid-19 Vaccine Candidates. *N Engl J Med* 2020; **383**: 2439–50.

5 Baden LR, El Sahly HM, Essink B, *et al.* Efficacy and Safety of the mRNA-1273 SARS-CoV-2 Vaccine. *New England Journal of Medicine* 2021; **384**: 403–16.

6 Anderson EJ, Rouphael NG, Widge AT, *et al.* Safety and Immunogenicity of SARS-CoV-2 mRNA-1273 Vaccine in Older Adults. *N Engl J Med* 2020; **383**: 2427–38.

7 Logunov DY, Dolzhikova IV, Shcheblyakov DV, *et al.* Safety and efficacy of an rAd26 and rAd5 vector-based heterologous prime-boost COVID-19 vaccine: an interim analysis of a randomised controlled phase 3 trial in Russia. *The Lancet* 2021; **397**: 671–81.

8 Logunov DY, Dolzhikova IV, Zubkova OV, *et al.* Safety and immunogenicity of an rAd26 and rAd5 vector-based heterologous prime-boost COVID-19 vaccine in two formulations: two open, non-randomised phase 1/2 studies from Russia. *The Lancet* 2020; **396**: 887–97.

9 Folegatti PM, Ewer KJ, Aley PK, *et al.* Safety and immunogenicity of the ChAdOx1 nCoV-19 vaccine against SARS-CoV-2: a preliminary report of a phase 1/2, single-blind, randomised controlled trial. *The Lancet* 2020; **396**: 467–78.

10 ANVISA. Announcement of results from Butantan Phase III clinical trial to evaluate efficacy and safety of the adsorbed (inactivated) COVID-19 vaccine produced by Sinovac.

11 Zhang Y, Zeng G, Pan H, *et al.* Safety, tolerability, and immunogenicity of an inactivated SARS-CoV-2 vaccine in healthy adults aged 18–59 years: a randomised, double-blind, placebo-controlled, phase 1/2 clinical trial. *The Lancet Infectious Diseases* 2021; **21**: 181–92.

12 Novavax. Announcement of UK and South Africa Trial Results. 2021; published online Jan 28. https://ir.novavax.com/events/event-details/novavax-update-conference-call-and-webcast-0 (accessed March 8, 2021).

13 Keech C, Albert G, Cho I, *et al.* Phase 1–2 Trial of a SARS-CoV-2 Recombinant Spike Protein Nanoparticle Vaccine. *N Engl J Med* 2020; **383**: 2320–32.

14 Janssen Biotech, Inc. FDA Briefing Document. Janssen Ad26.COV2.S Vaccine for the Prevention of COVID-19. Sponsor Briefing Document. 2021; published online Feb 26. https://www.fda.gov/media/146219/download (accessed March 8, 2021).

15 Sadoff J, Le Gars M, Shukarev G, *et al.* Interim Results of a Phase 1–2a Trial of Ad26.COV2.S Covid-19 Vaccine. *N Engl J Med* 2021; NEJMoa2034201.

16 Janssen Biotech, Inc. FDA Briefing Document. Janssen Ad26.COV2.S Covid-19 Vaccine for the Prevention of COVID-19. Sponsor Briefing Document Addendum. 2021; published online Feb 26. https://www.fda.gov/media/146218/download (accessed Feb 26, 2021).

17 Ramasamy MN, Minassian AM, Ewer KJ, *et al.* Safety and immunogenicity of ChAdOx1 nCoV-19 vaccine administered in a prime-boost regimen in young and old adults (COV002): a single-blind, randomised, controlled, phase 2/3 trial. *The Lancet* 2020; **396**: 1979–93.

18. Rohatgi, https://automeris.io/WebPlotDigitizer, 2020).
